# Calcium partitioning and allocation and blossom-end rot development in tomato plants in response to whole-plant and fruit-specific abscisic acid treatments

**DOI:** 10.1093/jxb/ert364

**Published:** 2013-11-12

**Authors:** Sergio Tonetto de Freitas, Andrew J. McElrone, Kenneth A. Shackel, Elizabeth J. Mitcham

**Affiliations:** ^1^Brazilian Agricultural Research Corporation, Embrapa Semiárido, Petrolina, PE, 56302-970, Brazil; ^2^USDA-ARS, Crops Pathology and Genetics Research Unit, Davis, CA 95616, USA; ^3^Department of Plant Sciences, University of California, Davis, CA 95616, USA

**Keywords:** ABA, apoplast, blossom-end rot, leakage, membrane, xylem sap.

## Abstract

The mechanisms regulating Ca^2+^ partitioning and allocation in plants and fruit remain poorly understood. The objectives of this study were to determine Ca^2+^ partitioning and allocation in tomato plants and fruit in response to whole-plant and fruit-specific abscisic acid (ABA) treatments, as well as to analyse the effect of changes in Ca^2+^ partitioning and allocation on fruit susceptibility to the Ca^2+^ deficiency disorder blossom-end rot (BER) under water stress conditions. Tomato plants of the cultivar Ace 55 (Vf) were grown in a greenhouse and exposed to low Ca^2+^ conditions during fruit growth and development. Starting 1 day after pollination (DAP), the following treatments were initiated: (i) whole plants were sprayed weekly with deionized water (control) or (ii) with 500mg l^−1^ ABA; or fruit on each plant were dipped weekly (iii) in deionized water (control) or (iv) in 500mg l^−1^ ABA. At 15 DAP, BER was completely prevented by whole-plant or fruit-specific ABA treatments, whereas plants or fruit treated with water had 16–19% BER incidence. At 30 DAP, BER was prevented by the whole-plant ABA treatment, whereas fruit dipped in ABA had a 16% and water-treated plants or fruit had a 36–40% incidence of BER. The results showed that spraying the whole plant with ABA increases xylem sap flow and Ca^2+^ movement into the fruit, resulting in higher fruit tissue and water-soluble apoplastic Ca^2+^ concentrations that prevent BER development. Although fruit-specific ABA treatment had no effect on xylem sap flow rates or Ca^2+^ movement into the fruit, it increased fruit tissue water-soluble apoplastic Ca^2+^ concentrations and reduced fruit susceptibility to BER to a lesser extent.

## Abstract (precis)

ABA prevented blossom-end rot development in tomato fruit by whole plant and fruit specific mechanisms. At the whole plant level, ABA increased fruit Ca^2+^ uptake. At the fruit specific level, ABA increased fruit tissue water-soluble apoplastic Ca^2+^ concentration

## Introduction

Calcium (Ca^2+^) is an essential plant nutrient required for proper plasma membrane function, in storage organelles to counterbalance anionic charges, in the cytosol for cellular signalling responses, and in the apoplast for cell wall structure (White and Broadley, 2003; Taylor and Locascio, 2004; Ho and White, 2005). Ca^2+^ deficiency disorders in fruit have been attributed to lower total tissue Ca^2+^ content, as well as abnormal regulation of cellular Ca^2+^ partitioning and distribution (Ho and White, 2005; Park *et al.*, 2005; De Freitas *et al.*, 2011a). The symptoms of Ca^2+^ deficiency disorders in fruit start with cell plasmolysis and the water-soaked appearance of blossom-end tissues that eventually becomes dark brown as cells die (Suzuki *et al.*, 2003; De Freitas *et al.*, 2010).

Although Ca^2+^ is believed to move in the plant exclusively through the xylem vascular tissue (Ho *et al.*, 1993; Taylor and Locascio, 2004), the mechanisms regulating Ca^2+^ partitioning and allocation in tomato plants and fruit remain poorly understood. Consistent with xylem sap flow, the direction and rate of xylemic Ca^2+^ flow in the plant should be determined by water potential gradients in response to different tissue transpiration and growth rates (Taylor and Locascio, 2004; Ho and White, 2005). In that case, higher transpiration and growth rates can reduce water potential and increase tissue strength as sinks for xylemic Ca^2+^. Therefore, the partitioning of Ca^2+^ flowing from the roots toward leaves and fruit will depend on the xylem sap Ca^2+^ concentration, as well as leaf and fruit transpiration and growth rates. Accordingly, leaves have much higher transpiration rates than fruit, which results in much higher Ca^2+^ content in the leaves than in the fruit (Ho, 1989; Ho and White, 2005; De Freitas *et al.*, 2011b).

Previous studies have shown that specifically reducing leaf transpiration by decreasing atmospheric vapour pressure deficit (VPD) or treating tomato plants with abscisic acid (ABA) can potentially decrease xylemic Ca^2+^ movement into the leaves, and increase its movement into the fruit (Guichard *et al.*, 2005; De Freitas *et al.*, 2011b). However, direct measurements of xylemic Ca^2+^ concentration and xylem sap flow rates into leaves and fruit in response to reduced leaf transpiration rates have not been reported.

Spraying whole plants with ABA increases fruit total tissue and apoplastic Ca^2+^ concentrations, and reduced fruit cell membrane leakage and the incidence of blossom-end rot (BER) (De Freitas *et al.*, 2011b). These studies suggest that ABA may affect not only total fruit tissue Ca^2+^ concentration but also the regulation of cellular Ca^2+^ distribution, which could affect fruit susceptibility to Ca^2+^ deficiency disorders such as BER (Park *et al.*, 2005; De Freitas *et al.*, 2011a, 2012). Since these studies were based on whole-plant ABA sprays, the results cannot be specifically attributed to whole-plant or fruit responses to ABA (De Freitas *et al.*, 2011b). Fruit-specific ABA studies are still needed to understand if the prevention of BER development is a whole-plant, a fruit-specific, or a combination response to ABA.

The objectives of this study were to determine Ca^2+^ partitioning and allocation in tomato plants and fruit in response to whole-plant and fruit-specific ABA treatments, as well as to analyse the effect of changes in Ca^2+^ partitioning and allocation on fruit susceptibility to BER under water stress conditions.

## Materials and methods

Tomato plants [*Solanum lycopersicum* cultivar Ace 55 (Vf)) were grown in 9.5 litre pots in a greenhouse environment with day/night temperature of 27 ºC/20 ºC and relative humidity of 64%/78%. The plants were grown in an organic substrate of equal portions of peat, sand, and redwood compost, with 2.6g kg^−1^ dolomite lime. Plants were irrigated once a day at about 08:00h with a nutrient solution containing N (102mg l^–1^), P (26mg l^–1^), K (124mg l^–1^), Ca (90mg l^–1^), Mg (24mg l^–1^), S (16mg l^–1^), Fe (1.6mg l^–1^), Mn (0.27mg l^–1^), Cu (0.16mg l^–1^), Zn (0.12mg l^–1^), B (0.26mg l^–1^), and Mo (0.016mg l^–1^). At full bloom, fully open flowers in the first cluster were tagged and manually pollinated. After pollination, plants were irrigated once a day at 08:00h with 450ml of the same nutrient solution, but without Ca^2+^, for the remaining period of the study to induce BER development in the fruit. The volume of nutrient solution added daily to the plants after pollination was determined from the volume of total plant water loss per day determined as described below (~450ml plant^–1^ d^–1^). This approach was used to induce plant hydration followed by plant dehydration in a 24h cycle. Wilting was typically observed in the control plants prior to irrigation in the morning, but plants recovered turgor upon irrigation. The water stress condition was used to enhance fruit susceptibility to BER.

Beginning 1 day after pollination (DAP), each plant was sprayed weekly with 200ml of deionized water (control) or 500mg l^−1^ ABA (Valent Biosciences, Libertyville, IL, USA), or 4–6 fruit on separate plants from those sprayed were dipped weekly in 300ml of deionized water (control) or 500mg l^−1^ ABA (Valent Biosciences). All solutions contained 0.5ml l^−1^ of Silwet L-77 (Helena Chemical Co., Collierville, TN, USA) as a surfactant. The dipping treatments were applied by completely immersing the fruit in each solution for a period of 10 s. All treatments were applied once a week until 30 DAP. Each treatment was composed of four single plant replications at each evaluation time. After evaluation, plants were eliminated from the experiment. On each plant, 6–8 flowers on the first cluster were tagged, resulting in 6–8 tagged fruit per replication. No thinning treatment was applied to non-tagged flowers or fruit. Plants and fruit were analysed at 15 and 30 DAP as described below. Fruit sampling was accomplished at the end of the irrigation cycle, before 08:00h.

### Relative humidity and air temperature

The relative humidity and air temperature inside the greenhouse were monitored daily every 15min. The results presented are the hourly averages from 15 to 30 DAP. VPD was calculated based on the hourly measured relative humidity and air temperature as described by Otieno *et al.* (2012).

### Percentage blossom-end rot incidence

The percentage BER was determined by dividing the number of tagged fruit with BER symptoms by the total number of tagged fruit per plant and multiplying this value by 100.

### Electrolyte leakage

Analysis of electrolyte leakage was carried out according to the method described by De Freitas *et al.* (2011a). Briefly, three fruit discs of 1cm diameter and 0.7cm thickness were cut from the blossom end of healthy fruit and sectioned with a double-bladed knife 1mm under the skin. Each sample of three discs from three fruit represented one replication, which was placed into a 50ml conical tube on a rotary shaker in a 0.2M mannitol solution. The conductivity of the mannitol solution was recorded periodically over 6h. Then, samples were frozen and thawed twice to determine the total conductivity (Saltveit, 2002). The results were expressed as the percentage increase in electrolyte leakage (conductivity) per gram of tissue per hour relative to the total tissue conductivity.

### Stem water potential (SWP)

The SWP was determined from 13:00h to 15:00h by bagging one leaf directly connected to the main stem in a reflective envelope for ~20min. Each bagged leaf was then cut from the plant and placed inside a pressure chamber (PMS Instrument Company, Albany, OR, USA) for SWP measurement (McCutchan and Shackel, 1992).

### Leaf stomatal conductance

This was analysed in two opposite, fully expanded leaves located on the base, middle, and top regions of each tomato plant. The base, middle, and top regions were defined by visually dividing the height of the plant into three equal sections. The stomatal conductance measurements were accomplished from 13:00h to 15:00h using a steady-state porometer (LI-1600; LI-COR Biotechnology, Lincoln, NE, USA).

### Total plant water loss

The total water loss was determined by irrigating the plants early in the morning. After draining, the pots were bagged and weighed. Twenty-four hours later, the pots were weighed again and the volume of water lost was calculated as the difference between the first and the last weight per plant.

### Xylem sap flow measurements

These were accomplished using the heat ratio method (HRM) with an external sap flow sensor (Burgess *et al.*, 2001; Green *et al.*, 2003; Clearwater *et al.*, 2009). The HRM was developed to measure low net sap flow rates that can take place in either direction in the vascular tissue (Burgess *et al.*, 2001; Green *et al.*, 2003; Clearwater *et al.*, 2009), but for the current study only the xylem sap flow rate was determined by heat girdling the middle leaf pedicel or fruit peduncle. The heat girdling was accomplished by passing an electrical signal (0.8 A, 10V) for 20 s across a constantan wire with 0.8mm diameter looped twice around the pedicel or peduncle 1cm upstream of each heat sensor before starting the sap flow measurements (Else *et al.*, 1996). Heat girdling destroys the phloem cells, obstructing phloem sap movement, while the xylem sap flow remains intact and functional due to its non-living cells. This technique has been used to isolate and quantify phloem and xylem sap flow rates (Else *et al.*, 1996; Guichard *et al.*, 2005; Peuke *et al.*, 2006; Clearwater *et al.*, 2009; Hossain and Nonami, 2010). After heat girdling, sap flow measurements were made over a 24h period. After xylem sap flow measurements, zero sap flow readings were determined by cutting the middle leaf pedicel or fruit peduncle 1cm downstream of each sensor. The zero xylem sap flow readings were used to determine the baseline accurately for each sap flow sensor after sap flow measurements. After determining the zero sap flow rate, the middle leaf pedicel or fruit peduncle was cut at the heat sensor region to measure the diameter of the xylem vascular tissue, which was used to calculate the volume of xylem sap moving into the leaf and fruit over time. One fully expanded top leaf and one tagged fruit on each plant replication were used for the sap flow analysis. All sensor signals were logged (CR10X, Campbell Scientific Inc., Logan, UT, USA) at 20min intervals and averaged every hour. The results presented are the averages of four replications. Xylem sap uptake (XSU) into the fruit was determined based on the daily average xylem sap flow measurements from 15 and 30 DAP.

### Extraction of soil solution, xylem sap, and apoplastic solution

Extractions were accomplished at the end of the irrigation cycle, before 08:00h. Soil solution was extracted by adding 450ml of the nutrient solution, without Ca^2+^, to each plant pot, and collecting the drained leachate. After collecting the soil leachate, plants were decapitated 15cm above the soil level and the pots containing the roots plus stem stump were used to extract the stem xylem sap. Two fruit and two middle leaves were harvested at the end of the irrigation cycle from each replicate plant with the entire peduncle and pedicel attached and kept in a sealed plastic bag for xylem sap extraction. Xylem sap extraction was accomplished by placing the plant pot, fruit, or leaf blade inside a pressure chamber (PMS Instrument Company), while the cut end of the stem, peduncle, or pedicel was exposed to the outside of the chamber through a seal. After sealing, the pressure inside the chamber was increased up to 0.8MPa with N_2_. The initial xylem sap moving out of the stem, peduncle, or pedicel cut end was blotted dry to reduce Ca^2+^ contamination from the cut. The following 100 μl was collected over a period of 5min and used to determine the Ca^2+^ concentration in the xylem sap (Wartinger *et al.*, 1990; Schurr and Schulze, 1995; Montanaro *et al.*, 2006). Fruit and leaf were pressurized inside a commercial pressure chamber (PMS Instrument Company). The plant pots were pressurized in a custom-built chamber large enough to hold a 9.5 litre pot, with a two-part (split) lid in order to allow assembly around the stem of an intact plant (PMS Instrument Company). The custom-built chamber was also used to pressurize the roots of whole plants to induce guttation on leaf blades, which were collected for Ca^2+^ quantification. The guttation samples represent the xylem sap extracted from leaf blades without any contamination from a cut surface. Apoplastic water-soluble Ca^2+^ was extracted from the blossom-end pericarp tissue of tomato fruit as previously described by De Freitas *et al.* (2011a).

### Calcium concentration

The Ca^2+^ concentration in the soil solution, xylem sap, and apoplastic solution was determined with an Ultra-M micro Ca^2+^-selective electrode (Lazar Research Laboratories, Inc., Los Angeles, CA, USA). A standard Ca^2+^ calibration curve (*R* ^2^=0.98) was used to determine the Ca^2+^ concentration in the samples.

The Ca^2+^ concentration in leaf and fruit tissues was determined in freeze-dried leaf blades, as well as pericarp tissues manually cut from the peduncle and blossom regions of the fruit. Freeze-dried samples were subjected to microwave acid digestion and analysed by inductively coupled plasma atomic emission spectrometry (Meyer and Keliher, 1992). Calcium accumulation was quantified by subtracting the total middle leaf and fruit Ca^2+^ contents observed at 15 DAP from the total middle leaf and fruit Ca^2+^ contents observed at 30 DAP. Calcium accumulation was also estimated by multiplying the quantified xylem sap Ca^2+^ concentration observed in the middle leaf pedicel and fruit peduncle by its respective daily average xylem sap flow rate observed at 15 and 30 DAP.

### Xylem function

This was measured in developing fruit as previously described by Ho *et al.* (1993) and De Freitas *et al.* (2011b). Fruits were harvested and held in sealed plastic bags for 20min with 100ml of free water to reduce transpiration until the peduncle of each fruit was immersed in a solution of 1% Safranin-O at 20 ºC under ≤20% relative humidity. After 24h, fruit were cut into three equal sections at a 90 º angle to the peduncle axis. The number of stained vascular bundles (xylem vessels) was counted in the placenta and pericarp tissues at the cut surfaces at the blossom and peduncle end regions of each fruit.

### Fruit growth rate

The rate of fruit growth was measured over a 24h period using dendrometers (Model DEX20, Dynamax Inc., Houston, TX, USA). One tagged fruit on each plant replication was used for fruit growth rate analysis. All sensor signals were logged (CR1000, Campbell Scientific Inc.) at 10min intervals and averaged every hour.

### Fruit weight

The fruit weight was determined by dividing the total weight of all tagged fruit on the plant by the total number of tagged fruit on each plant replication. The final result represents the average of all replications in each treatment. The average fruit water uptake for growth (WUG) was determined by subtracting the fruit water content quantified at 15 DAP from the fruit water content quantified at 30 DAP and dividing that value by 15 d. The results are presented as volume of sap uptake per fruit per day.

### Phloem sap uptake (PSU)

Analysis of the PSU was based on the assumption that the total fruit water uptake from 15 to 30 DAP was supplied only by xylem and phloem. Therefore, PSU was determined as the sum of WUG plus transpiration (Ts) minus XSU. In this case,


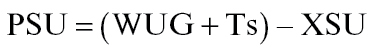


Based on previous studies, Ts=375 μl of water fruit^–1^ d^–1^, for the same tomato fruit developmental stage (Liu *et al.*, 2007). The values obtained for the calculations represent the average from 15 to 30 DAP. The results were expressed as μl of sap uptake fruit^–1^ d^–1^. Phloem sap solutes concentration (PSC) was calculated as the fruit dry weight (DW) accumulated from 15 to 30 DAP plus the carbon (C) weight loss due to fruit respiration (CWR) divided by the PSU from 15 to 30 DAP (Liu *et al.*, 2007; Génard and Lescourret, 2012). In this case,


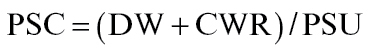


According to previous studies, CWR=0.0895g C fruit^–1^ d^–1^ for the same tomato fruit developmental stage (Liu *et al.*, 2007).

### Statistical analysis

Analysis of variance (ANOVA) was performed for each variable using the Statistical Analysis System (SAS) software package. The mean values were compared by Tukey’s test (*P*=0.05) or presented as means ±standard deviation (SD).

## Results

**Fig. 1.**
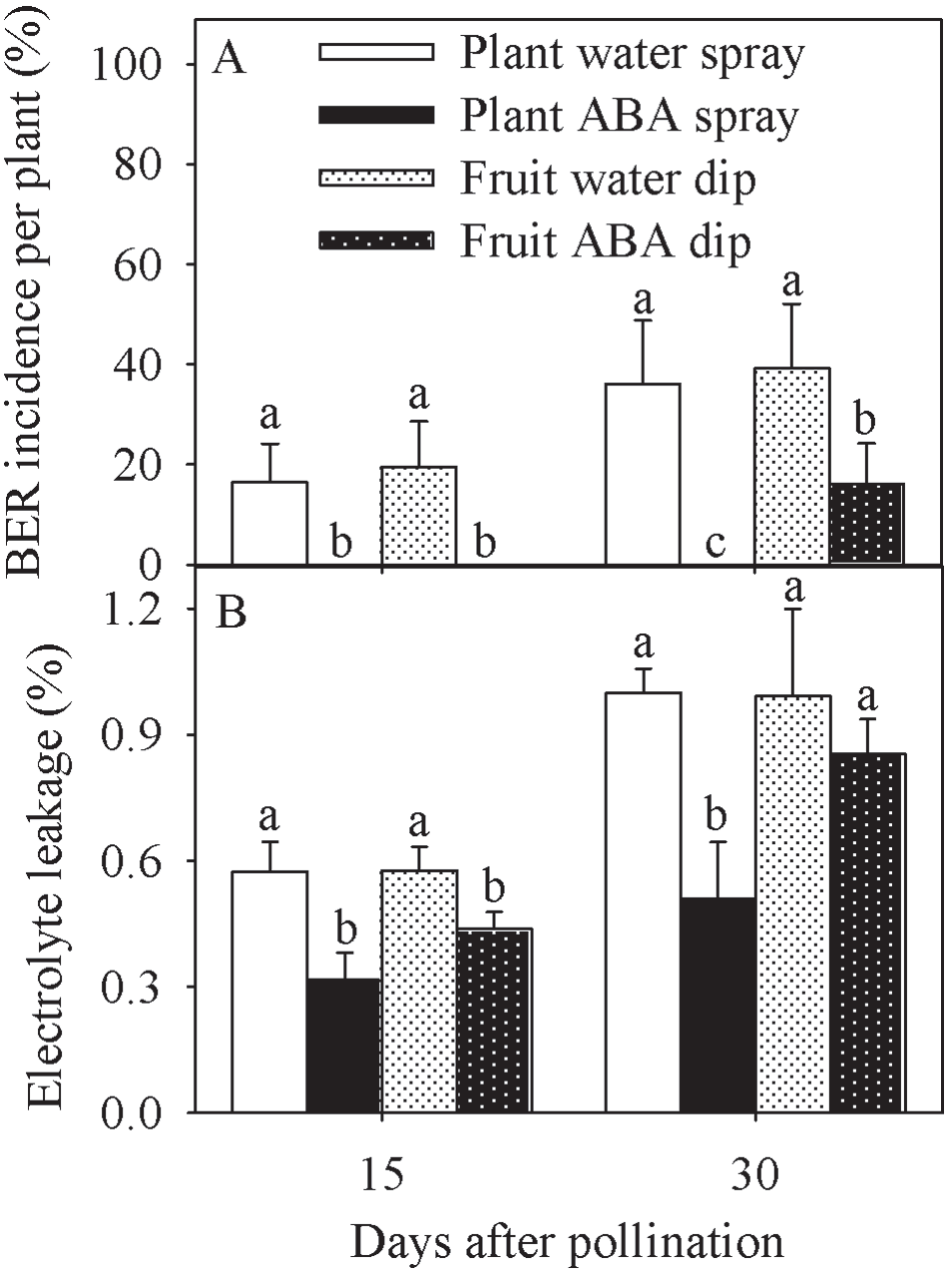
Blossom-end rot incidence (A) and electrolyte leakage (B) of tomato fruit of the Ace 55 (Vf) cultivar at 15 and 30 days after pollination (DAP). Vertical bars indicate means ±SD, *n*=4 for all treatments. Means with the same letter at each evaluation time are not significantly different (*P*=0.05) according to Tukey’s test.

BER was completely suppressed by spraying the whole plants weekly with ABA during fruit growth and development, compared with water-sprayed fruit that reached a 36% incidence of BER at 30 DAP (Fig. 1A). Dipping the fruit in solutions containing ABA prevented BER development at 15 DAP, but ABA-dipped fruit reached a 16% incidence of BER at 30 DAP. Control fruit dipped in water had a 39% incidence of BER at 30 DAP. The electrolyte leakage of fruit pericarp tissue was lower in response to whole-plant and fruit-specific ABA treatments at 15 DAP (Fig. 1B). At 30 DAP, only the whole-plant ABA treatment had lower electrolyte leakage in fruit pericarp tissue.

**Fig. 2.**
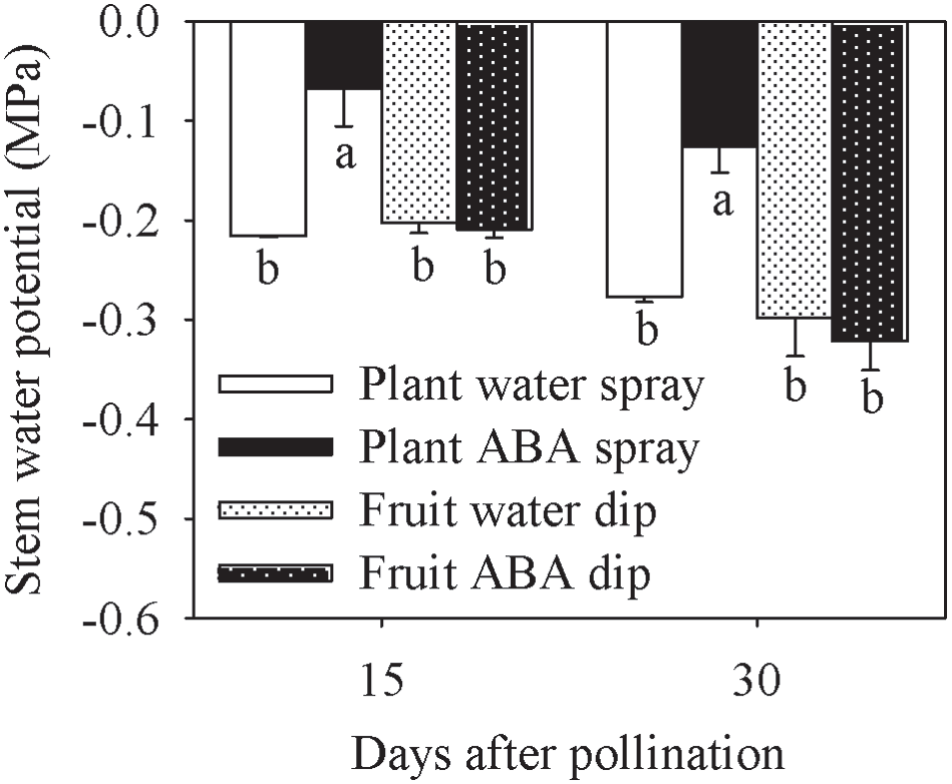
Midday stem water potential (SWP) of tomato plants of the Ace 55 (Vf) cultivar at 15 and 30 DAP. Vertical bars indicate means ±SD, *n*=4 for all treatments. Means with the same letter at each evaluation time are not significantly different (*P*=0.05) according to Tukey’s test.

**Fig. 3.**
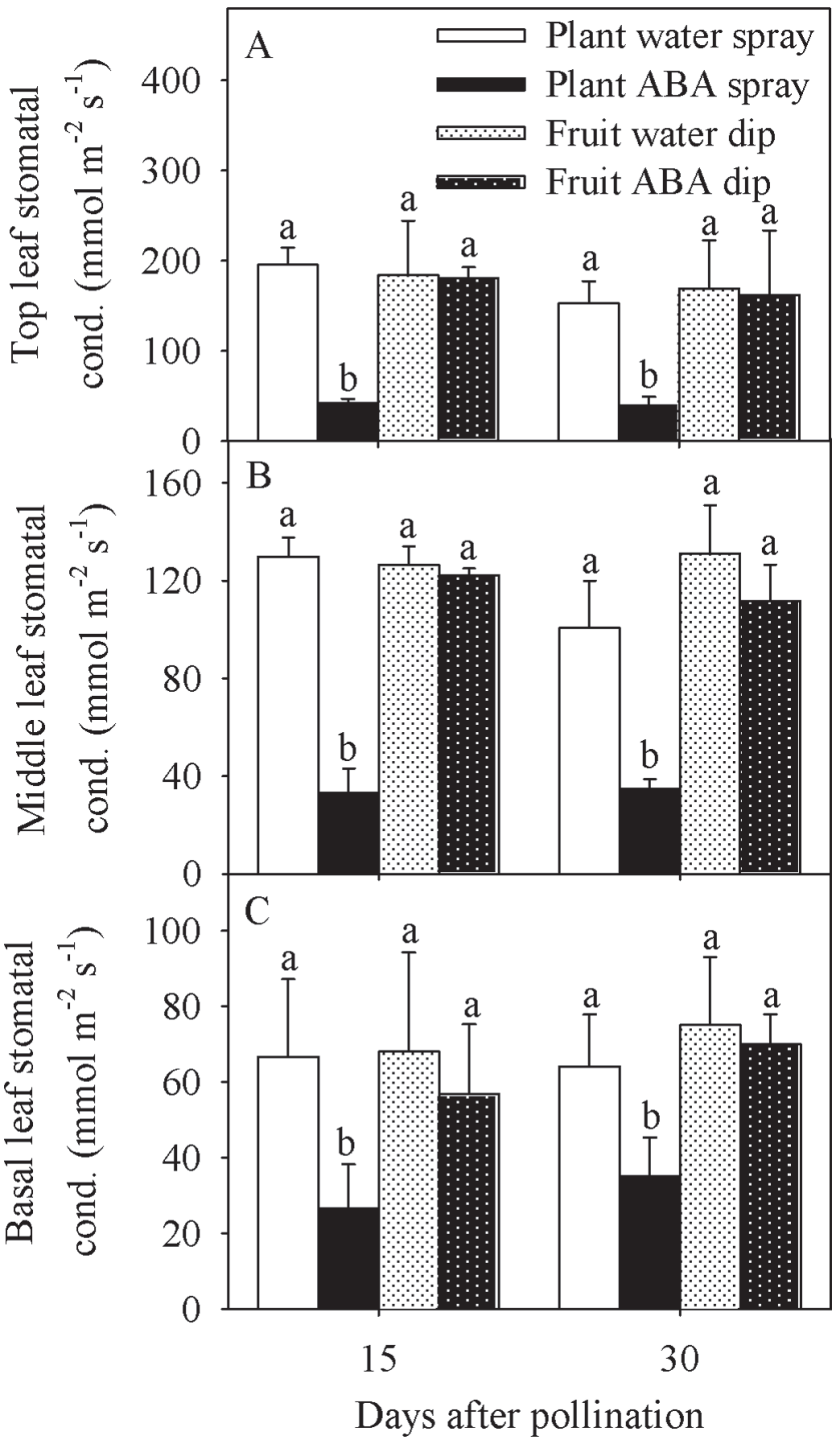
Top (A), middle (B), and basal (C) leaf stomatal conductance of tomato plants of the Ace 55 (Vf) cultivarat 15 and 30 DAP. Vertical bars indicate means ±SD, *n*=4 for all treatments. Means with the same letter at each evaluation time are not significantly different (*P*=0.05) according to Tukey’s test.

SWP was less negative in response to whole-plant ABA treatment at 15 and 30 DAP compared with all other treatments (Fig. 2). Leaf stomatal conductance progressively increased from the base, middle, to the top regions of the plant, and was lower in the whole-plant ABA treatment at 15 and 30 DAP compared with all other treatments (Fig. 3). Whole-plant water spraying, as well as water and ABA dip treatments, had similar stomatal conductance for the basal, middle, and top leaves. Based on the stomatal conductance analysis, the plant ABA uptake was considered high with whole-plant ABA treatment, and no significant ABA movement from the fruit into the plant was observed based on changes in stomatal conductance in response to fruit-specific ABA dip treatment (Fig. 3).

**Fig. 4.**
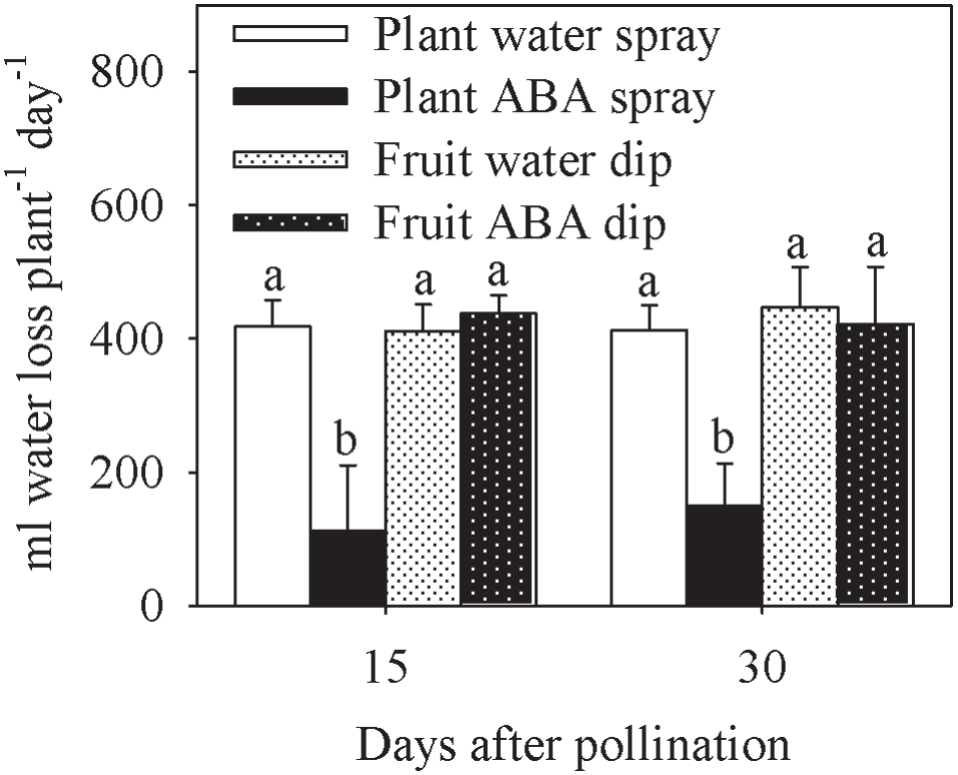
Daily water loss of tomato plants of the Ace 55 (Vf) cultivar at 15 and 30 DAP. Vertical bars indicate means ±SD, *n*=4 for all treatments. Means with the same letter at each evaluation time are not significantly different (*P*=0.05) according to Tukey’s test.

**Fig. 5.**
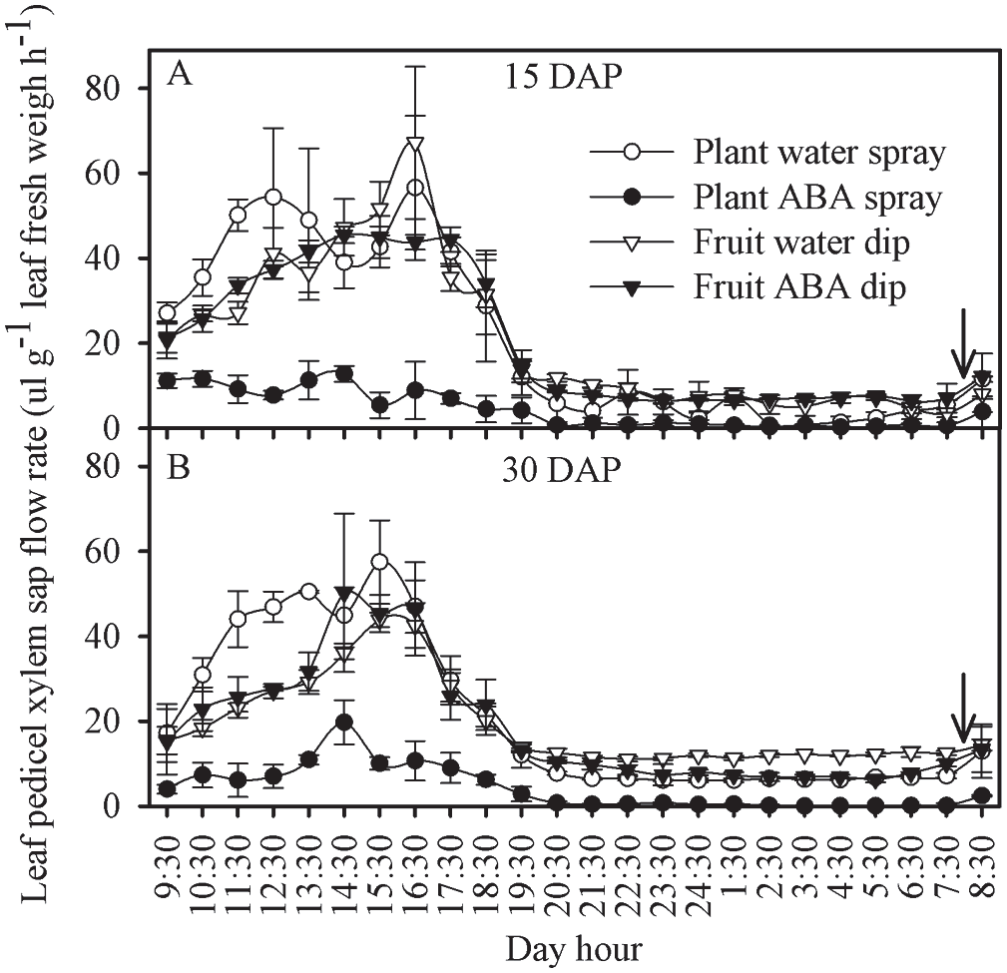
Sap flow rate in the top leaf pedicel of tomato plants of the Ace 55 (Vf) cultivar at 15 (A) and 30 (B) DAP. Vertical bars indicate means ±SD, *n*=4 for all treatments. The arrow represents the time of irrigation.

Plant water loss was reduced by the whole-plant ABA treatment at 15 and 30 DAP (Fig. 4), but was similar in all other treatments, including the ABA fruit dip. Whole-plant ABA treatment maintained a significantly lower xylem sap flow rate into the top leaves compared with all other treatments, especially during the daylight hours over a 24h period at 15 and 30 DAP (Fig. 5, Table 1). The water spray treatment had the highest sap flow during most of the daylight period at 15 and 30 DAP (Fig. 5).

**Table 1.** Average xylemic sap flow rate in leaf pedicel and fruit peduncle of tomato cultivar Ace 55 (Vf) at 15 and 30 DAP

Treatment	Leaf pedicel (μl g^–1^ leaf FW h^–1^)		Fruit peduncle (μl g^–1^ fruit FW h^–1^)	
	**15 DAP**	**30 DAP**	**15 DAP**	**30 DAP**
Plant water spray	20.7 a*	20.6 a	0.34 b	0.09 c
Plant ABA spray	4.4 b	4.2 b	1.27 a	0.27 a
Fruit water dip	20.5 a	19.0 a	0.33 b	0.08 c
Fruit ABA dip	20.2 a	18.2 a	0.36 b	0.16 b
CV (%)	19.8	18.2	14.6	5.4

*Means with the same letter between treatments at each evaluation time are not significantly different (*P*=0.05) according to Tukey’s test.

CV, coefficient of variation.

**Fig. 6.**
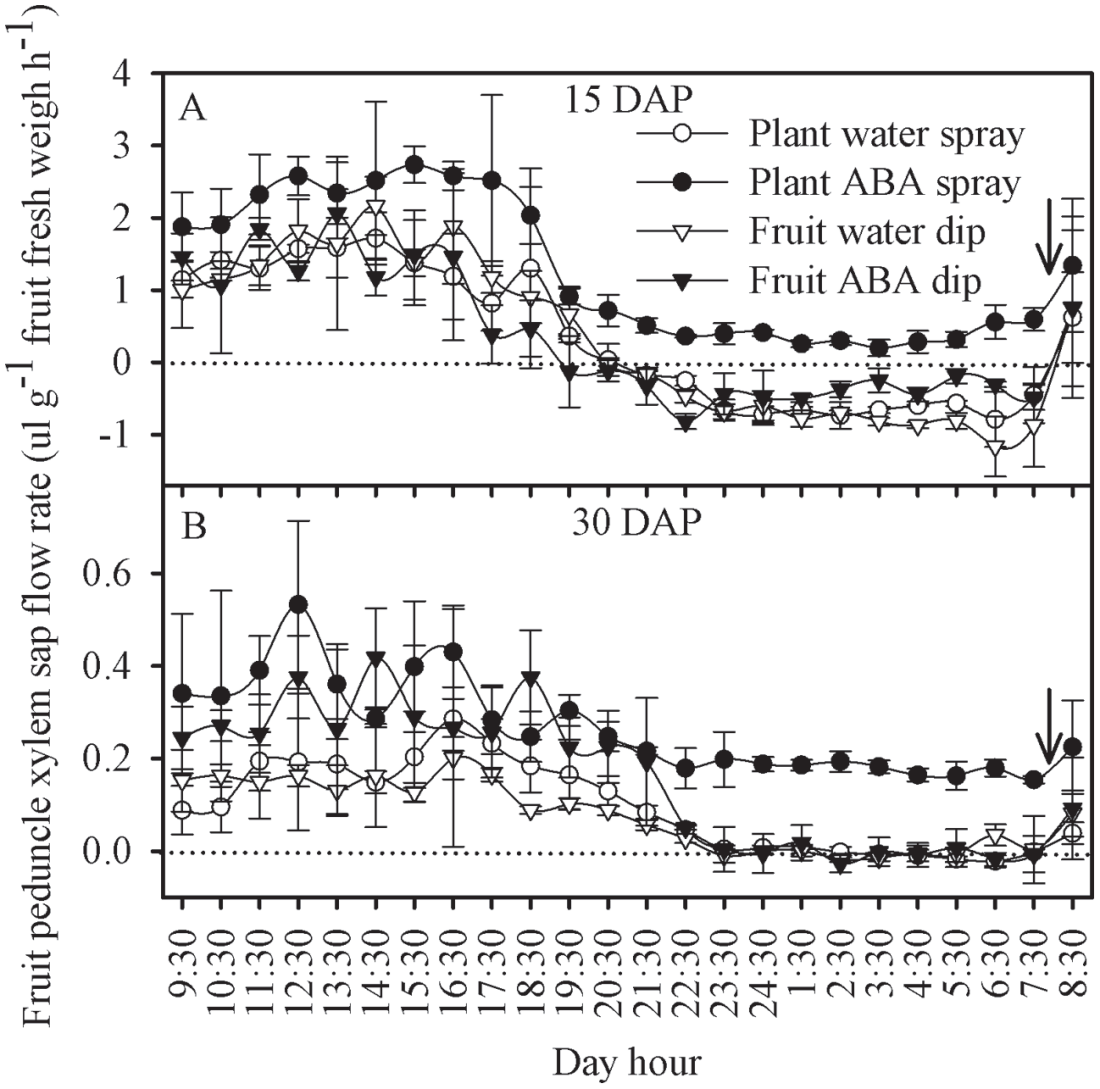
Sap flow rate in fruit peduncle of tomato cultivar Ace 55 (Vf) at 15 (A) and 30 (B) DAP. Vertical bars indicate means ±SD, *n*=4 for all treatments. The arrow represents the time of irrigation. Note the difference in the *y*-scale.

The average flow of xylem sap moving into the fruit during a 24h irrigation cycle was substantially higher on plants sprayed with ABA, compared with all other treatments (Fig. 6, Tables 1, 2), with the same diurnal pattern as seen in the leaves (Fig. 5). At 15 DAP, fruit on water-sprayed plants, as well as water- and ABA-dipped fruit had a reverse flow of xylemic sap from the fruit back to the plant, starting in the late afternoon until the next irrigation cycle in the morning (Fig. 6A). The ABA-dipped treatment had a slightly higher sap flow to the fruit than the two water treatments at 15 and at 30 DAP (Table 1). Fruit on plants sprayed with ABA had no reverse xylemic sap flow throughout the irrigation cycle at 15 DAP, but had the same diurnal pattern, with the lowest flows occurring during the night period (Fig. 6A). The diurnal pattern of fruit xylem flow at 30 DAP was similar to that at 15 DAP, but the magnitude was substantially reduced, again with no discernible reverse flow (Fig. 6B, Tables 1, 2). Spraying tomato plants with ABA resulted in higher total fruit water uptake used for growth and lower fruit water uptake through the phloem from 15 to 30 DAP (Table 2). The estimated phloem sap solute concentration uptake into the fruit from 15 to 30 DAP was higher in ABA-sprayed plants than in non-sprayed plants (Table 2).

**Table 2.** Average fruit water uptake for growth, fruit water uptake through the xylem and phloem vessels, and phloem solute concentration from 15 to 30 DAP

Treatment	Growth water uptake (μl fruit^–1^ d^–1^)	Xylem sap uptake (μl fruit^–1^ d^–1^)	Phloem sap uptake (μl fruit^–1^ d^–1^)	Phloem solutes (mg ml^–1^)
Plant water spray	964 b*	222 b	1117 a	132 b
Plant ABA spray	1450 a	1046 a	778 b	301 a
Fruit water dip	800 b	189 b	986 a	146 b
Fruit ABA dip	902 b	259 b	1018 a	155 b
CV (%)	11.5	10.2	10.8	10.9

*Means with the same letter between treatments at each evaluation time are not significantly different (*P*=0.05) according to Tukey’s test.

CV, coefficient of variation.

The Ca^2+^ concentrations in the soil solution and in the main stem xylem sap were similar among all treatments at 15 and 30 DAP. The average Ca^2+^ concentration in the soil solution among treatments was 1.41±0.09mM at 15 DAP and 1.08±0.14mM at 30 DAP. The average Ca^2+^ concentration in the main stem xylem sap was 0.72±0.04mM at 15 DAP and 0.63±0.05mM at 30 DAP. There was no statistical difference among treatments in Ca^2+^ concentrations in the xylem sap of basal, middle, or top leaves at 15 or 30 DAP. The same results were obtained when Ca^2+^ was determined on an independent set of plants under the same treatments using the leaf guttation method (data not shown). The average xylem sap Ca^2+^ concentrations were 0.71±0.06mM and 0.86±0.02mM in top leaves, 0.77±0.03mM and 0.87±0.02mM in middle leaves, and 0.64±0.03mM and 0.81±0.02mM in basal leaves at 15 and 30 DAP, respectively. The Ca^2+^ concentration in the peduncle xylem sap was higher in fruit from ABA-sprayed plants at 15 and 30 DAP, compared with all other treatments (Table 3), and water-soluble apoplastic Ca^2+^ was higher in fruit from ABA-sprayed plants at 15 and 30 DAP, compared with all other treatments (Table 3). Fruit dipped in ABA solution had slightly higher water-soluble apoplastic Ca^2+^ than fruit dipped in water and fruit from plants sprayed with water at 15 DAP.

**Table 3.** Calcium concentration in the xylem sap of fruit peduncle and in the apoplast of blossom-end pericarp tissue of the tomato fruit Ace 55 (Vf) cultivar at 15 and 30 DAP

Treatment	Fruit peduncle xylem Ca^2+^ (mM)		Fruit blossom-end apoplastic Ca^2+^ (mM)	
	**15 DAP**	**30 DAP**	**15 DAP**	**30 DAP**
Plant water spray	0.73 b*	1.00 b	0.04 c	0.13 b
Plant ABA spray	0.97 a	1.24 a	0.27 a	0.46 a
Fruit water dip	0.71 b	0.87 b	0.04 c	0.11 b
Fruit ABA dip	0.71 b	0.86 b	0.09 b	0.18 b
CV (%)	12.3	11.8	24.2	27.5

*Means with the same letter between treatments at each evaluation time are not significantly different (*P*=0.05) according to Tukey’s test.

CV, coefficient of variation.

**Fig. 7.**
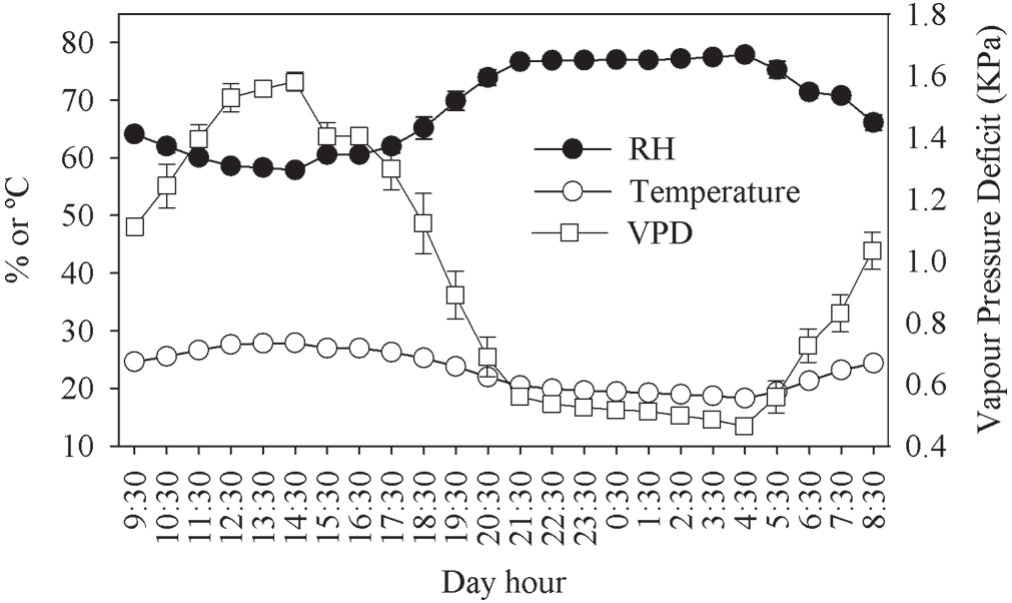
Average air relative humidity (RH), temperature, and vapour pressure deficit (VPD) in the greenhouse environment during growth and developm ent of cultivar Ace 55 (Vf) tomatoes from 15 to 30 DAP. Vertical bars indicate means ±SD.

The Ca^2+^ concentration in the top and middle leaves was statistically lower in response to whole-plant ABA treatment compared with all other treatments at 15 and 30 DAP (Table 6). The Ca^2+^ concentrations in ABA-sprayed plants were 8.7±0.21 and 8.1±0.09mg g DW^–1^ in top leaves and 17.5±0.52 and 16.1±0.63mg g DW^–1^ in middle leaves at 15 and 30 DAP, respectively. The Ca^2+^ concentrations in all other non-ABA-sprayed plants were 13.0±0.36 and 13.0±0.15mg g DW^–1^ in top leaves and 25.1±0.96 and 23.9±0.81mg g DW^–1^ in middle leaves at 15 and 30 DAP, respectively. The Ca^2+^ concentration in basal leaves was similar in all treatments at 15 DAP (average=36.7±1.5mg g DW^–1^), and statistically lower in plants sprayed with ABA (average=27.4±0.42mg g DW^–1^) than all other treatments (average=32.8±0.53mg g DW^–1^) at 30 DAP. The Ca^2+^ concentration in fruit tissue collected at the peduncle and blossom ends of the fruit was higher in ABA-sprayed plants at 15 and 30 DAP (Table 4). Fruit dipped in ABA had a higher Ca^2+^ concentration at the blossom-end tissue at 15 DAP, compared with water-dipped fruit and fruit of water-sprayed plants (Table 4). Ca^2+^ accumulation was lower in the leaf and higher in the fruit of ABA-sprayed plants than in the other treated plants and fruit from 15 to 30 DAP (Table 5). Ca^2+^ accumulation in leaf and fruit quantified by tissue analysis was similar to the estimated Ca^2+^ accumulation based on the Ca^2+^ concentration in the xylem sap and xylem sap flow rates into leaf and fruit tissues (Table 5). The average relative humidity and air temperature from 15 to 30 DAP inside the greenhouse, where the tomato plants were grown, oscillated from 58.2% and 27.8 °C during the day up to 77.8% and 18.2 °C during the night, respectively (Fig. 7). The VPD increased from 0.5 kPa at 05:30h to 1.6 kPa at 14:30h, decreasing thereafter (Fig. 7).

**Table 4.** Calcium concentration observed in the peduncle end and blossom-end tissues of tomato fruit of the Ace 55 (Vf) cultivar at 15 and 30 DAP

Treatment	Fruit peduncle end (mg Ca^2+^ g DW^–1^)		Fruit blossom end (mg Ca^2+^ g DW^–1^)	
	**15 DAP**	**30 DAP**	**15 DAP**	**30 DAP**
Plant water spray	0.74 b*	0.70 b	0.34 c	0.25 b
Plant ABA spray	1.41 a	1.14 a	0.80 a	0.59 a
Fruit water dip	0.66 b	0.59 b	0.30 c	0.26 b
Fruit ABA dip	0.69 b	0.57 b	0.48 b	0.25 b
CV (%)	8.7	18.7	8.6	11.4

*Means with the same letter between treatments at each evaluation time are not significantly different (*P*=0.05) according to Tukey’s test.

CV, coefficient of variation.

**Table 5.** Quantified (QCA) and estimated (ECA) Ca^2+^ accumulation in leaf and fruit of the tomato Ace 55 (Vf) cultivar from 15 to 30 DAP

Treatment	μg Ca^2+^ leaf^–1^ d^–1^			μg Ca^2+^ fruit^–1^ d^–1^		
	QCA	ECA^*a*^	Ratio^*b*^	QCA	ECA^*a*^	Ratio^*b*^
Plant water spray	203.4 a*	201.6 a	0.99	8.3 b	7.1 b	0.85
Plant ABA spray	40.3 b	39.9 b	0.99	49.8 a	43.1 a	0.86
Fruit water dip	197.7 a	195.8 a	0.99	7.1 b	5.6 b	0.79
Fruit ABA dip	196.6 a	186.7 a	0.94	9.1 b	7.9 b	0.86
CV (%)	8.8	13.6		11.8	11.0	

*Means with the same letter between treatments are not significantly different (*p*=0.05) according to Tukey’s test.

^*a*^Calcium accumulation was estimated by multiplying the xylem sap Ca^2+^ concentration observed in middle leaf pedicel and fruit peduncle by its respective xylem sap flow rate.

^*b*^ Ratio=estimated/quantified.

CV, coefficient of variation.

**Fig. 8.**
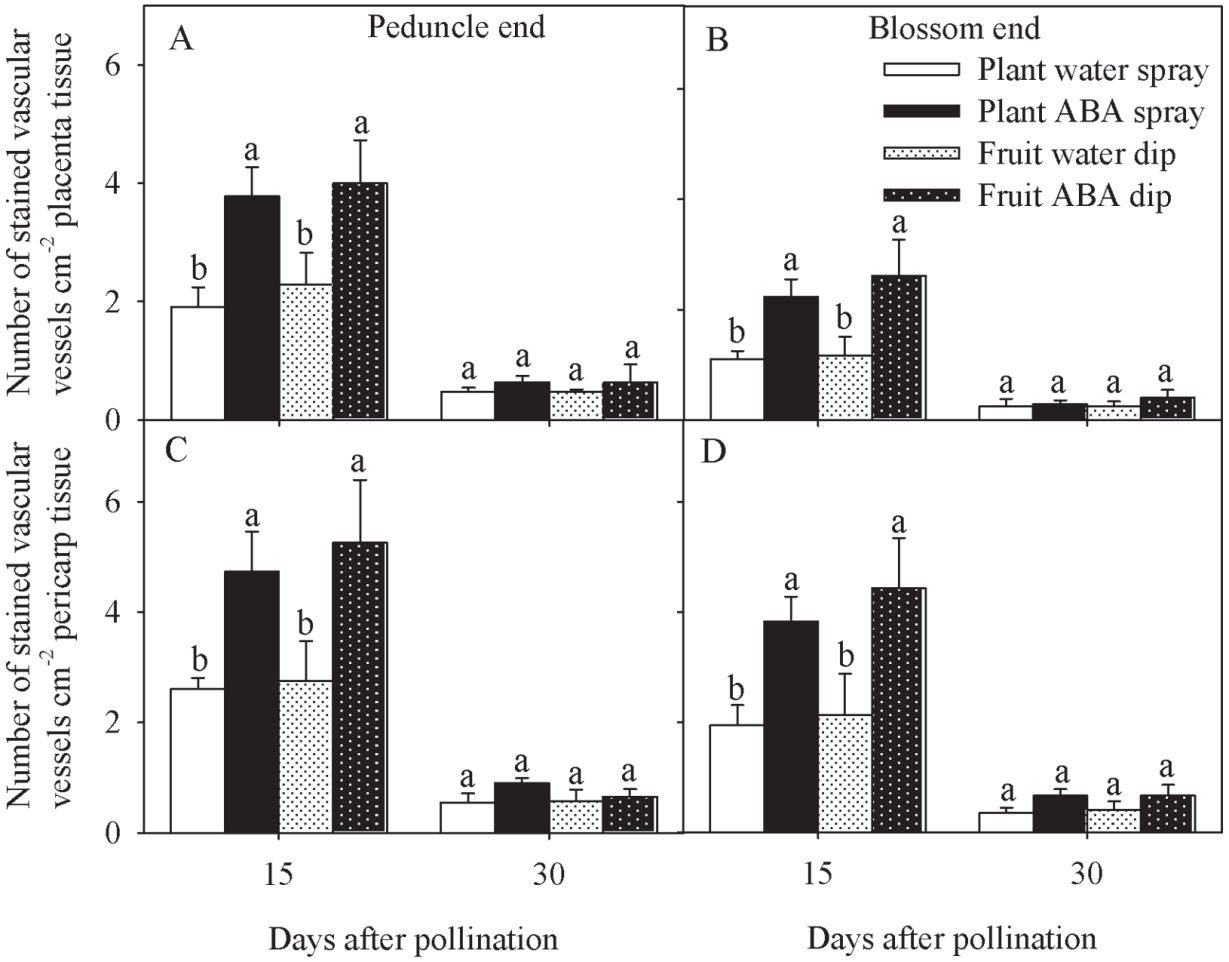
Number of stained vascular bundles in the placenta (A and B) and pericarp (C and D) tissues at the peduncle (A and C) and blossom-end regions (B and D) of Ace 55 (Vf) tomato fruit. Vascular bundles were stained with 1% Safranin-O. Vertical bars indicate means ±SD, *n*=4 for all treatments. Means with the same letter at each evaluation time are not significantly different (*P*=0.05) according to Tukey’s test.

The number of Safranin-O-stained vascular bundles in the placenta and pericarp tissues at the peduncle and blossom-end regions of the fruit was higher in response to whole-plant and fruit-specific ABA treatments at 15 DAP (Fig. 8). The number of stained vascular bundles decreased in all treatments from 15 to 30 DAP, and all treatments showed a similar number of stained vascular bundles in the placenta and pericarp tissues at the peduncle and blossom-end regions of the fruit at 30 DAP (Fig. 8).

**Fig. 9.**
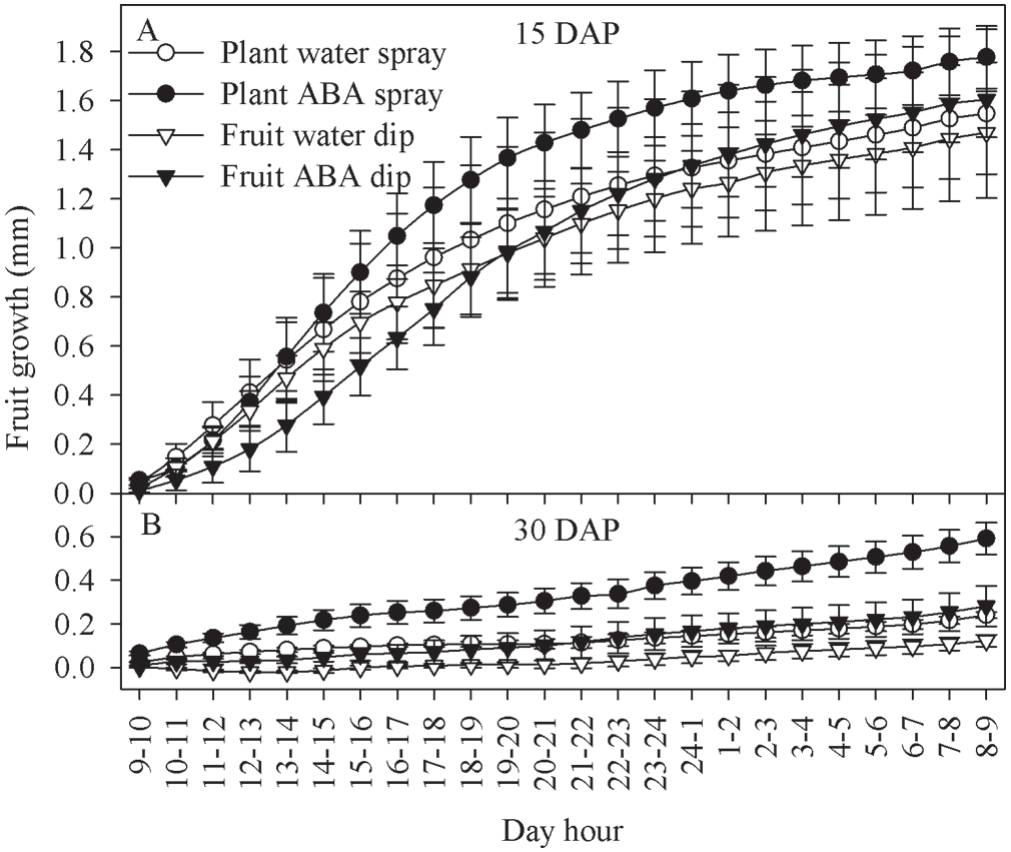
Growth rate of tomato fruit of the Ace 55 (Vf) cultivar at 15 and 30 DAP. Vertical bars indicate means ±SD, *n*=4 for all treatments.

**Fig. 10.**
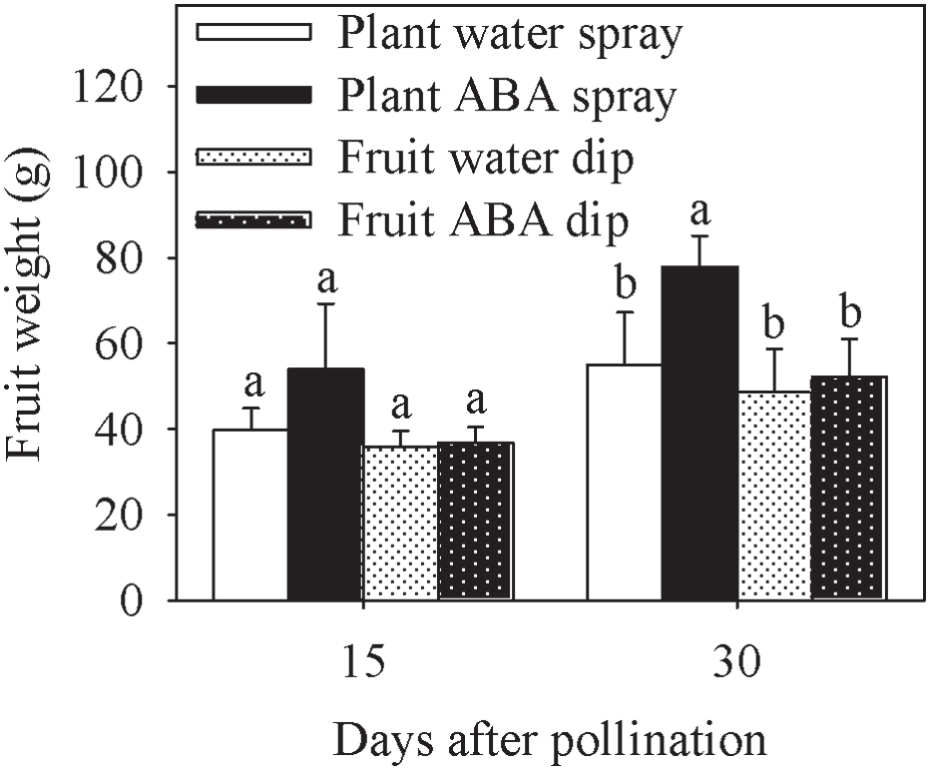
Average weight of tomato fruit of the Ace 55 (Vf) cultivar at 15 and 30 DAP. Vertical bars indicate means ±SD, *n*=4 for all treatments. Means with the same letters at each evaluation time are not significantly different (*P*=0.05) according to Tukey’s test.

The fruit growth rate was higher in ABA-sprayed plants compared with all other treatments at 15 and 30 DAP (Fig. 9). All treatments showed a positive fruit growth rate during a 24h period at 15 and 30 DAP (Fig. 9). For all treatments, the fruit growth rate was higher at 15 DAP than at 30 DAP (Fig. 9). The average fruit weight was also higher in ABA-sprayed plants at 15 and 30 DAP (Fig. 10). Fruit Ca^2+^ uptake, both directly quantified and estimated based on the product of fruit xylem sap uptake and fruit peduncle xylem sap Ca^2+^ concentration, was 6-fold higher in ABA-sprayed plants compared with water-sprayed controls (Table 6). A much smaller increase in Ca^2+^ uptake was found in ABA-dipped fruit, but, again, this was consistent for both directly quantified and estimated values (Table 6). The sprayed and dipped ABA/water ratios for fruit growth rate were 1.41 and 1.15, respectively (Table 6).

**Table 6.** Ratios between sprayed or dipped ABA and sprayed or dipped water treatments for quantified Ca^2+^ accumulation (QCA), xylem sap uptake (XSU), Ca^2+^ concentration in the fruit peduncle xylem sap (CFXS), estimated Ca^2+^ accumulation (ECA), and growth rate of tomato fruit of the Ace 55 (Vf) cultivar from 15 to 30 DAP

Analysis	Plant spray			Fruit dip		
	Water	ABA	Ratio^*a*^	Water	ABA	Ratio
1. QCA (μg fruit^–1^ d^–1^)	8.30 b*	49.80 a	6.00	7.10 a	9.10 a	1.28
2. XSU (μl fruit^–1^ d^–1^)	221.5 b	1046.4 a	4.72	189.0 a	259.2 a	1.37
3. CFXS (mM)^*b*^	0.86 b	1.10 a	1.28	0.79 a	0.78 a	1.00
4. ECA^*c*^ (μg fruit^–1^ d^–1^)	–	–	6.04	–	–	1.37
5. Fruit growth (mm d^–1^)	1.27 b	1.80 a	1.41	1.07 a	1.23 a	1.15

*Means with the same letter within spray or dip treatments were not significantly different (*P*=0.05) according to Tukey’s test

^*a*^ Ratio=ABA/water.

^b^ CFXS=average Ca^2+^ concentration in the fruit peduncle xylem sap between 15 and 30 DAP.

^*c*^ Estimated Ca^2+^ accumulation (ECA)=Line 2×Line 3.

## Discussion

Previous studies showed that weekly spraying of tomato plants with ABA prevented BER development in the fruit, while water-sprayed plants reached a 30–45% incidence of BER at 40–45 DAP (De Freitas *et al.*, 2011b). At that time, possible mechanisms through which ABA increased fruit Ca^2+^ concentration and reduced fruit susceptibility to BER were suggested based on estimations of fruit xylem sap uptake and Ca^2+^ concentration in the xylem sap (De Freitas *et al.*, 2011b). In the present study, direct measurements of xylem sap uptake and xylem sap Ca^2+^ concentration in the main stem, leaf pedicel, and fruit peduncle were used to demonstrate that xylem sap flow and Ca^2+^ movement into fruit were substantially increased with spraying the whole plant with ABA.

### Whole-plant Ca^2+^ uptake and partitioning in response to ABA

Water uptake in leaves comes exclusively from xylem vessels, while water uptake into the fruit comes from both phloem and xylem vascular tissues (Ho and White, 2005). Treating the whole plant with ABA reduced stomatal conductance, which resulted in lower plant water loss, lower soil water uptake and xylemic water movement into the leaves, as well as higher SWP and increased xylemic water movement into the fruit. Considering that Ca^2+^ concentrations in the soil solution and stem xylem sap were similar among all treatments, the observed lower Ca^2+^ accumulation in ABA-sprayed plants was due to lower soil solution uptake triggered by lower leaf transpiration rates (Table 5). Our results also estimate a higher solute concentration in the phloem sap moving into the fruit of ABA-sprayed plants (Table 2). Although ABA reduced stomatal conductance and this would be expected to decrease leaf photosynthesis (Egea *et al.*, 2011), the improved plant water status associated with ABA application may have caused compensatory physiological effects in other areas, such as reduced carbon partitioning to roots and/or improved carbon transport rates, resulting in higher solute concentration in the phloem sap, compared with the other treatments. The non-ABA-sprayed plants had an average fruit phloem sap uptake of 1.04ml fruit^–1^ d^–1^ and an average phloem sap solute concentration of 144.3mg ml^–1^ (Table 2). The ABA-sprayed plants had an average fruit phloem sap uptake of 0.778ml fruit^–1^ d^–1^ and an average phloem sap solute concentration of 301mg ml^–1^ (Table 2). Therefore, the solute accumulation per day was 1.04×144.3=150.07mg fruit^–1^ d^–1^ in non-ABA-sprayed plants and 0.778×301=234.18mg fruit^–1^ d^–1^ in ABA-sprayed plants. Therefore, the results also showed that ABA-sprayed plants had higher phloem solute accumulation per fruit than non-ABA-sprayed plants from 15 to 30 DAP. According to the present data and other studies, ABA could also be acting at the whole-plant level as a signal triggering carbohydrate accumulation and osmotic adjustment in sink organs (Alves and Setter, 2004). In addition, spraying peach fruit with ABA has been shown to increase the activity of sorbitol oxidase, a predominant enzyme in the metabolism of the translocated sugar alcohol sorbitol, which was followed by an enhanced sugar accumulation in the fruit (Kobashi *et al.*, 1999). The higher phloem sap solute concentration in ABA-sprayed plants can decrease fruit apoplastic solute potential, which is then equilibrated by a parallel decline in fruit total water potential (Matthews *et al.*, 1987). Under these conditions, higher fruit solute accumulation can increase the water potential gradient between the fruit and stem, favouring fruit xylem sap uptake (Johnson *et al.*, 1992; Taylor and Locascio, 2004; Ho and White, 2005). Accordingly, the present results show that whole-plant ABA spray treatment not only increased phloem solute accumulation per fruit, but also decreased leaf transpiration, maintaining a higher stem water potential and higher total fruit water uptake, compared with non-ABA-sprayed plants.

### Leaf xylem sap and Ca^2+^ uptake in response to ABA

Following irrigation in the morning, tomato plants treated with ABA had a smaller increase in xylem sap flow rate into the leaves compared with non-treated plants, presumably due to suppression of stomatal opening (Neill *et al.*, 2008). In all treatments, the increase in xylem sap flow after the time of irrigation in the morning probably reflected the combined effects of leaf rehydration as well as increasing light intensity stimulating stomatal opening, and increasing VPD from increased air temperatures and decreased relative humidity in the greenhouse environment. The reduction in leaf xylem sap flow after 15:30h to 16:30h was presumably a result of the reverse changes in the environmental conditions observed early in the day after irrigation. These results and other studies also show a direct relationship between high leaf transpiration and higher leaf Ca^2+^ uptake (De Freitas *et al.*, 2011b), suggesting that leaf Ca^2+^ accumulation is dependent only on leaf xylem sap uptake triggered by leaf transpiration rates. In addition, the data also show that Ca^2+^ concentration in the leaf xylem sap extracted by pressurizing the leaves or by inducing leaf guttation were similar, suggesting that there is no significant Ca^2+^ contamination of leaf xylem sap when the leaves are cut and pressurized in the pressure chamber for xylem sap extraction.

### Fruit xylem sap and Ca^2+^ uptake in response to ABA

The ABA-treated fruit also showed a higher number of stained xylem vessels (potentially functional vessels). In that case, ABA treatment may have also reduced the hydraulic resistance within the fruit, favouring xylemic water movement in the fruit towards the blossom-end tissue, provided a hydrostatic gradient responsible for xylem sap flow was present in the fruit (Bondada *et al.*, 2005). Since Ca^2+^ is believed to be mobile in the plant exclusively through the xylem vessels (Ho and White, 2005), the observed increase in xylem sap flow towards the fruit in the pedicle, and a reduced hydraulic resistance within the fruit, may explain the observed higher fruit Ca^2+^ accumulation in ABA-treated plants. Neither effect was observed in ABA-dipped fruit, suggesting that changes in Ca^2+^ partitioning in the plant are responsive only to whole-plant ABA treatment.

The pattern of fruit xylem sap uptake followed an increase after irrigation in all treatments until 15:30h to 16:30h, decreasing thereafter at both 15 and 30 DAP. Similar to the leaves, this pattern could be explained by the combined effects of an increase in plant water content right after irrigation and an increase in VPD early during the day that increased the evaporative demand due to increasing air temperatures and decreasing relative humidity durig the day time. Late in the day, decreasing SWP due to plant water loss and decrease in the VPD and consequently the evaporative demand due to decreasing air temperatures and increasing relative humidity could limit xylem sap flow into the fruit, resulting in the observed decrease in fruit xylem sap uptake. At night, the VPD was low but not zero, and continued plant water loss under these conditions may have been associated with the observed reverse flow of fruit xylem sap at 15 DAP for non-ABA-treated plants. Although a reverse xylem sap flow was observed later in the irrigation cycle, the fruit growth rate was always positive, indicating that phloem sap uptake maintained the positive growth rates even under reverse xylem sap flow conditions. The reverse flow of fruit xylem sap was not observed at 30 DAP, possibly because of higher fruit solute content compared with 15 DAP. The higher solute content decreased fruit water potential (Bolarin *et al.*, 2001), which possibly increased the strength of the fruit as a sink for xylemic sap uptake under limited xylem conductivity conditions. The present results showed that fruit xylem sap uptake decreased from 15 to 30 DAP in tomato. Previous studies have shown that phloem may represent 76–83% and xylem may represent 17–24% of fruit peduncle water uptake at early stages of growth and development (Tables 3, 4; De Freitas *et al.*, 2011b). Consistent with the data presented, other studies have also shown that at later stages of growth and development, the xylem contribution to fruit water uptake decreases due to loss of xylem functionality and/or reduction in the hydrostatic gradient responsible for xylemic sap uptake and movement in the fruit (Ho *et al.*, 1993; Dražeta *et al.*, 2004; Bondada *et al.*, 2005; De Freitas *et al.*, 2011b). However, other studies have shown that xylem transport into trusses of tomato fruit cultivar Gourmet remained functional throughout the first 8 weeks of growth. In addition, these studies showed that ~75% of water net influx into the fruit occurred through the external xylem and ~25% via the perimedullary region, which contains both phloem and xylem (Windt *et al.* 2009). Differences in the phloem/xylem ratio of fruit sap uptake presented in the literature could be attributed to different genotypes and/or thegrowing conditions of each study. In future studies, direct measurements of phloem sap uptake into the fruit using nuclear magnetic resonance should be carried out for the same tomato cultivar and growing conditions as used in the present study to compare precisely the methods and the results obtained (Windt *et al.* 2009).

Although no statistically significant changes in Ca^2+^ concentrations in stem xylem sap were observed among the treatments, spraying plants with ABA increased the Ca^2+^ concentration in the xylem sap moving into the fruit. The movement of Ca^2+^ in the xylem vessels depends on adsorption and desorption of Ca^2+^ from active exchange sites within the cell walls (McLaughlin and Wimmer, 1999; Taylor and Locascio, 2004). In that case, fruit of ABA-sprayed plants possibly had exchange sites within the xylem cell walls that were more saturated with Ca^2+^, maintaining higher levels of soluble Ca^2+^ in the xylem sap stream. In addition, evidence suggests that special nutrient transport systems exist at the interface between living cells and xylem vessels (De Boer and Volkov, 2003). The higher Ca^2+^ concentration observed in the xylem sap of the peduncle of fruit from ABA-sprayed plants could be the result of the higher flow rate of xylem sap into the fruit leading to a higher saturation of Ca^2+^ binding sites in the xylem vessels and cell uptake requirements that reduced Ca^2+^ binding to active exchange sites in the cell walls as well as the Ca^2+^ uptake into living cells at the interface with the xylem vessels.

### Calcium concentration in the fruit in response to ABA

Spraying tomato plants with ABA increased the Ca^2+^ concentration and Ca^2+^ accumulation in the pericarp tissue at the fruit peduncle end by increasing fruit xylem sap uptake, decreasing fruit phloem sap uptake, increasing Ca^2+^ concentration in the xylem sap moving into the fruit, and possibly by increasing phloem Ca^2+^ transport into the fruit.

The results show that ABA spray treatment increased fruit xylem sap uptake4.72-fold, fruit xylem sap Ca^2+^ concentration 1.28-fold, and fruit growth 1.41-fold, compared with water spray treatment, respectively. These results suggest that the increase in fruit xylem sap uptake was the most important effect of ABA spray treatment leading to the observed higher fruit Ca^2+^ accumulation from 15 to 30 DAP. The Ca^2+^ accumulation in fruit tissue estimated by multiplying the xylem sap Ca^2+^ concentration in the fruit peduncle by its respective flow rate into the fruit from 15 to 30 DAP was ~84% (average among treatments) of the Ca^2+^ accumulation quantified by the difference in total fruit Ca^2+^ content observed at 30 DAP minus the total fruit Ca^2+^ content observed at 15 DAP. Considering that fruit water uptake is via the xylem and phloem, the results suggest that the phloem may have also contributed to fruit Ca^2+^ uptake under the experimental conditions described in this study. The results also show a greater difference between the quantified and estimated Ca^2+^ accumulation in the fruit of ABA-sprayed plants (6.7 μg Ca^2+^ fruit^–1^ d^–1^) than in the fruit of other treatments (1.2–1.5 μg Ca^2+^ fruit^–1^ d^–1^), suggesting that spraying plants with ABA also enhanced fruit phloem Ca^2+^ uptake. Considering that spraying tomato plants with ABA decreased fruit phloem sap uptake, it is possible that this treatment increased Ca^2+^ concentration in the phloem sap to increase fruit Ca^2+^ uptake to compensate for the reduction of phloem sap uptake. These results agree with previous studies suggesting that phloem can also have an important contribution to fruit Ca^2+^ uptake depending on the phloem sap Ca^2+^ concentration and phloem sap flow rate into the fruit (Jones *et al.*, 1983, 1986; Jones and Samuelson, 1983; De Freitas *et al.*, 2011b). In the present study, it was assumed that fruit transpiration rates were similar among all treatments. Future studies related to the effect of ABA on xylem and phloem fruit water uptake should include direct measurements of fruit transpiration rates.

In the xylem vessels, after reaching the peduncle end of the fruit, Ca^2+^ can be taken up by the cells, bind to active exchange sites within the cell walls, or remain soluble in the xylem vessels to be translocated towards the blossom-end tissues of the fruit (McLaughlin and Wimmer, 1999; De Boer and Volkov, 2003; Taylor and Locascio, 2004). Accordingly, the present results show that higher xylem sap and tissue Ca^2+^ content at the fruit peduncle end resulted in higher fruit Ca^2+^ translocation to and Ca^2+^ accumulation in the blossom-end tissues in response to whole-plant ABA treatment.

Dipping the fruit in ABA did not affect xylem sap or tissue Ca^2+^ content at the fruit peduncle end, but resulted in higher Ca^2+^ accumulation and higher Ca^2+^ in the apoplast in the blossom-end tissue at 15 DAP, suggesting that ABA also triggered a fruit-specific mechanism that favoured Ca^2+^ translocation from the peduncle end towards the blossom-end region of the fruit. This latter effect was not observed at 30 DAP. According to the present data, spraying the whole plant with ABA or dipping the fruit in ABA maintained a higher number of functional xylem vessels that reduced the resistance to xylemic water and Ca^2+^ movement into the blossom-end tissue, which could help to explain the observed higher Ca^2+^ content in the distal end of the fruit. In ABA-dipped fruit, the increase in Ca^2+^ concentration in the blossom-end tissue was only observed at 15 DAP, possibly due to the reduction in any ABA effect on maintaining a higher number of functional xylem vessels at late stages of fruit growth and development. It is possible that ABA could also increase the number of functional xylem vessels connecting the fruit to the plant, which should be determined in future studies. In addition, higher cuticular wax content in epidermal cells at 30 DAP compared with fruit at 15 DAP (Leide *et al.*, 2007) could limit fruit ABA uptake during the later dip treatments.

### Possible mechanisms controlling fruit susceptibility to BER at the whole-plant and fruit-specific levels in response to ABA

At the whole-plant level, ABA treatment triggered stomatal closure, decreasing xylemic water and Ca^2+^ flow to the leaves, which maintained higher stem water potential. Under such conditions, whole-plant ABA treatment favoured xylemic water and Ca^2+^ movement into the rapidly expanding fruit, resulting in higher Ca^2+^ content reaching the fruit peduncle end. However, the data suggest that xylem sap uptake could not fully explain fruit Ca^2+^ accumulation due to the difference between the observed total fruit Ca^2+^ accumulation and the estimated fruit Ca^2+^ accumulation based on the Ca^2+^ concentration in the xylem sap and xylem sap flow rate into the fruit. These results suggest that phloem could have acted as a source of Ca^2+^ to the fruit under the experimental conditions described in this study. More detailed studies should include direct measurements of fruit transpiration rates to better characterize the role of phloem in fruit Ca^2+^ uptake. In addition, a better understanding of phloem contributions to fruit Ca^2+^ uptake can be accomplished by developing efficient methods to extract and quantify Ca^2+^ in the phloem sap moving into the fruit.

At the fruit-specific level, ABA maintained a higher number of functional xylem vessels at early stages of growth and development, reducing the resistance to Ca^2+^ movement in the fruit and thus allowing Ca^2+^ to be translocated towards the blossom-end tissue. Such Ca^2+^ accumulation in the blossom-end tissue was enhanced in response to whole-plant ABA treatment, compared with fruit-specific ABA treatment, possibly due to the additional effects on increasing xylem sap flow rate into the fruit, and Ca^2+^ concentration in the xylem sap taken up by the fruit.

Blossom-end tissue has the lowest concentration of Ca^2+^ in the fruit, and for that reason is the most susceptible tissue in the fruit to Ca^2+^ deficiency disorders (Taylor and Locascio, 2004; Ho and White, 2005). Studies suggest that Ca^2+^ deficiency symptoms are triggered by a depletion of the apoplastic pool of Ca^2+^ required to bind to phospholipids and proteins on the plasma membrane (White and Broadley, 2003; Taylor and Locascio, 2004; Ho and White, 2005). Under conditions of low apoplastic Ca^2+^, the plasma membrane can become leaky, leading to cell plasmolysis and eventually death (Suzuki *et al.*, 2003; De Freitas *et al.*, 2011a). Accordingly, the results show that higher Ca^2+^ accumulation in the blossom-end tissue in response to ABA treatment resulted in higher water-soluble apoplastic Ca^2+^ concentration, lower membrane leakage, and reduced fruit susceptibility to BER development. In this context, new tomato cultivars with higher ABA biosynthesis could be selected not only for water saving purposes, but also for reduced fruit susceptibility to BER. However, it is possible that fruit water content will be higher in high ABA genotypes, which may reduced the post-harvest life of fresh fruit.

## Abbreviations:

ABA: abscisic acid
BER: blossom-end rot
C: carbon
Ca^2+^: calcium
CFXS: Ca^2+^ concentration in the fruit peduncle xylem sap
CWR: carbon weight loss due to respiration
DAP: day after pollination
DW: dry weight
ECA: estimated Ca^2+^ accumulation
HRM: heat ratio method
PSC: phloem sap solutes concentration
PSU: phloem sap uptake
QCA: quantified Ca^2+^ accumulation
SWP: stem water potential
Ts: transpiration
VPD: vapour pressure deficit
WUG: water uptake for growth
XSU: xylem sap uptake.

## References

[CIT0001] Alves AAC Setter TL 2004 Abscisic acid accumulation and osmotic adjustment in casava under water deficit. Environmental and Experimental Botany 51, 259–271

[CIT0002] Bolarin MC Estan MT Caro M Romero-Aranda R Cuartero J 2001 Relationship between tomato fruit growth and fruit osmotic potential under salinity. Plant Science 160, 1153–1159 11337072 10.1016/s0168-9452(01)00360-0

[CIT0003] Bondada BR Matthews MA Shackel KA 2005 Functional xylem in the post-veraison grape berry. Journal of Experimental Botany 56, 2949–2957 16207748 10.1093/jxb/eri291

[CIT0004] Burgess SSO Adams MA Turner NC Beverly CR Ong CK Khan AAH Bleby TM 2001 An improved heat pulse method to measure low and reverse rates of sap in wood plants. Tree Physiology 21, 589–598 11390303 10.1093/treephys/21.9.589

[CIT0005] Clearwater MJ Luo Z Mazzeo M Dichio B 2009 An external heat pulse method for measurement of sap flow through fruit pedicels, leaf petioles and other small-diameter stems. Plant, Cell and Environment 32, 1652–1663 10.1111/j.1365-3040.2009.02026.x 19671100

[CIT0006] De Boer AH Volkov V 2003 Logistics of water and salt transport through the plant: structure and functioning of the xylem. Plant, Cell and Environment 26, 87–101

[CIT0007] De Freitas ST Amarante CVT Labavitch JM Mitcham E 2010 Cellular approach to understand bitter pit development in apple fruit. Postharvest Biology and Technology 57, 6–13

[CIT0008] De Freitas ST Jiang CZ Mitcham EJ 2012 Mechanisms involved in calcium deficiency development in tomato fruit in response to gibberellins. Journal of Plant Growth Regulation 31, 221–234

[CIT0009] De Freitas ST Padda M Wu Q Park S Mitcham E 2011a Dynamic alterations in cellular and molecular components during blossom-end rot development in tomatoes expressing *sCAX1*, a constitutively active Ca^2+^/H^+^ antiporter from Arabidopsis. Plant Physiology 156, 844–855 21464475 10.1104/pp.111.175208 PMC3177280

[CIT0010] De Freitas ST Shackel KA Mitcham EJ 2011b Abscisic acid triggers whole-plant and fruit-specific mechanisms to increase fruit calcium uptake and prevent blossom end rot development in tomato fruit. Journal of Experimental Botany 62, 2645–2656 21282326 10.1093/jxb/erq430

[CIT0011] Dražeta L Lang A Hall AJ Volz RK Jameson PE 2004 Causes and effects of changes in xylem functionality in apple fruit. Annals of Botany 93, 275–282 14988096 10.1093/aob/mch040 PMC4242201

[CIT0012] Egea G Verhoef A Vidale PL 2011 Towards an improved and more flexible representation of water stress in coupled photosynthesis–stomatal conductance models. Agricultural Forest Meteorology 151, 1370–1384

[CIT0013] Else MA Tiekstra AE Croker SJ Davies WJ Jackson MB 1996 Stomatal closure in flooded tomato plants involves abscisic acid and a chemically unified anti-transpirant in xylem sap. Plant Physiology 112, 239–247 12226387 10.1104/pp.112.1.239 PMC157942

[CIT0014] Génard M Lescourret F 2012 Using SWAF, a generic biophysical model of sugar and water accumulation to analyze fruit development. Acta Horticulturae 932, 203–212

[CIT0015] Green S Clothier B Jardine B 2003 Theory and practical application of heat pulse to measure sap flow. Agronomy Journal 95, 1371–1379

[CIT0016] Guichard S Gary C Leonardi C Bertin N 2005 Analysis of growth and water relations of tomato fruit in relation to air vapor pressure deficit and plant fruit load. Journal of Plant Growth Regulation 24, 201–213

[CIT0017] Ho LC 1989 Environmental effects on the diurnal accumulation of ^45^Ca by young fruit and leaves of tomato plants. Annals of Botany 63, 281–288

[CIT0018] Ho LC Belda R Brown M Andrews J Adams P 1993 Uptake and transport of calcium and the possible causes of blossom-end rot in tomato. Journal of Experimental Botany 44, 509–518

[CIT0019] Ho LC White PJ 2005 A cellular hypothesis for the induction of blossom-end rot in tomato fruit. Annals of Botany 95, 571–581 15642726 10.1093/aob/mci065 PMC4246855

[CIT0020] Hossain MM Nonami H 2010 Effect of water flow from the xylem on the growth-induced water potential and the growth-effective turgor associated with enlarging tomato fruit. Environmental Control in Biology 48, 101–116

[CIT0021] Johnson RW Dixon MA Lee DR 1992 Water relations of the tomato during fruit growth. Plant, Cell and Environment 15, 947–953

[CIT0022] Jones HG Higgs KH Samuelson TJ 1983 Calcium uptake by developing apple fruits. I. Seasonal changes in calcium content of fruits. Journal of Horticultural Science 58, 173–182

[CIT0023] Jones HG Higgs KH Samuelson TJ 1986 Calcium uptake by developing apple fruits: III. Additional studies on fruit calcium balance. Journal of Horticultural Science 61, 171–179

[CIT0024] Jones HG Samuelson TJ 1983 Calcium uptake by developing apple fruits. II. The role of spur leaves. Journal of Horticultural Science 58, 183–190

[CIT0025] Kobashi K Gemma H Iwahori S 1999 Sugar accumulation in peach (*Prunus persica*) fruit as affected by abscisic acid (ABA) treatment in relation to some sugar metabolizing enzymes. Journal of the Japanese Society for Horticultural Science 68, 465–470

[CIT0026] Leide J Hildebrandt U Reussing K Riederer M Vogg G 2007 The developmental pattern of tomato fruit wax accumulation and its impact on cuticular transpiration barrier properties: effects of a deficiency in a β-ketoacyl-coenzyme A synthase (LeCER6). Plant Physiology 144, 1667–1679 17468214 10.1104/pp.107.099481 PMC1914139

[CIT0027] Liu HF Genard M Guichard S Bertin N 2007 Model-assisted analysis of tomato fruit growth in relation to carbon and water fluxes. Journal of Experimental Botany 58, 3567–3580 18057037 10.1093/jxb/erm202

[CIT0028] Matthews MA Cheng G Weinbaum SA 1987 Changes in water potential and dermal extensibility during grape berry development. Journal of the American Society for Horticultural Science 112, 314–319

[CIT0029] McCutchan H, Shackel KA 1992 Stem water potential as a sensitive indicator of water stress in prune trees (Prunus domestica L. cv. French). Journal of the American Society for Horticultural Science 117, 607–611

[CIT0030] McLaughlin SB Wimmer R 1999 Tansley review No. 104. Calcium physiology and terrestrial ecosystem processes. New Phytologist 142, 373–417

[CIT0031] Meyer GA Keliher PN 1992 An overview of analysis by inductively coupled plasma-atomic emission spectrometry. In: Montaser A Golightly DW, eds. Inductively coupled plasmas in analytical atomic spectrometry. New York: VCH Publishers, 473–516

[CIT0032] Montanaro G Dichio B Xiloyannis C Celano G 2006 Light influences transpiration and calcium accumulation in fruit of kiwifruit plants (*Actinidia deliciosa* var. *deliciosa*). Plant Science 170, 520–527

[CIT0033] Neill S Barros R Bright J Desikan R Hancock J Harrison J Morris P Ribeiro D Wilson I 2008 Nitric oxide, stomatal closure, and abiotic stress. Journal of Experimental Botany 59, 165–176 18332225 10.1093/jxb/erm293

[CIT0034] Otieno D Lindner S Muhr J Borken W 2012 Sensitivity of peatland herbaceous vegetation to vapor pressure deficit influences net ecosystem CO_2_ exchange. Wetlands 32, 895–905

[CIT0035] Park S Cheng NH Pittman JK Yoo KS Park J Smith RH Hirschi KD 2005 Increasing calcium levels and prolonged shelf live in tomatoes expressing Arabidopsis H^+^/Ca^2+^ transporters. Plant Physiology 139, 1194–1206 16244156 10.1104/pp.105.066266 PMC1283758

[CIT0036] Peuke AD Windt C Van As H 2006 Effect of cold-girdling on flows in the transport phloem in *Ricinus communis*: is mass flow inhibited? Plant, Cell and Environment 29, 15–25 10.1111/j.1365-3040.2005.01396.x 17086749

[CIT0037] Saltveit ME 2002 The rate of ion leakage from chilling-sensitive tissue does not immediately increase upon exposure to chilling temperatures. Postharvest Biology and Technology 26, 295–304

[CIT0038] Schurr U Schulze ED 1995 The concentration of xylem sap constituents in root exudate, and in sap from intact, transpiring castor bean plants (*Ricinus communis* L.). Plant, Cell and Environment 18, 409–420

[CIT0039] Suzuki K Shono M Egawa Y 2003 Localization of calcium in the pericarp cells of tomato fruit during the development of blossom-end rot. Protoplasma 222, 149–156 14714203 10.1007/s00709-003-0018-2

[CIT0040] Taylor MD Locascio SJ 2004 Blossom-end rot: a calcium deficiency. Journal of Plant Nutrition 27, 123–139

[CIT0041] Wartinger A Heilmeier H Hartung W Schulze ED 1990 Daily and seasonal courses of leaf conductance and abscisic acid in the xylem sap of almond trees (*Prunus dulcis* (Miller) D.A. Webb) under desert conditions. New Phytologist 16, 581–587 10.1111/j.1469-8137.1992.tb00051.x 33874050

[CIT0042] White PJ Broadley MR 2003 Calcium in plants. Annals of Botany 92, 487–511 12933363 10.1093/aob/mcg164 PMC4243668

[CIT0043] Windt CW Gerkema E Van As H 2009 Most water in the tomato truss is imported through the xylem, not the phloem: a nuclear magnetic resonance flow imaging study. Plant Physiology 151, 830–842 19710234 10.1104/pp.109.141044 PMC2754649

